# Testing for non-linear causal effects using a binary genotype in a Mendelian randomization study: application to alcohol and cardiovascular traits

**DOI:** 10.1093/ije/dyu187

**Published:** 2014-09-05

**Authors:** Richard J Silverwood, Michael V Holmes, Caroline E Dale, Debbie A Lawlor, John C Whittaker, George Davey Smith, David A Leon, Tom Palmer, Brendan J Keating, Luisa Zuccolo, Juan P Casas, Frank Dudbridge

**Affiliations:** ^1^Faculty of Epidemiology and Population Health, ^2^Centre for Statistical Methodology and ^3^Bloomsbury Centre for Genetic Epidemiology and Statistics, London School of Hygiene and Tropical Medicine, London, UK, ^4^Department of Epidemiology and Public Health, University College London, London, UK, ^5^MRC Integrative Epidemiology Unit and ^6^School of Social and Community Medicine, University of Bristol, Bristol, UK, ^7^Genetics, R & D, GlaxoSmithKline, Stevenage, UK, ^8^Division of Health Sciences, University of Warwick, Warwick, Coventry, UK, ^9^Center for Applied Genomics, Abramson Research Center, Children's Hospital of Philadelphia, Philadelphia, PA, USA and ^10^Institute of Cardiovascular Science, University College London, London, UK

**Keywords:** Mendelian randomization, instrumental variables, causal inference, local average treatment effects, alcohol consumption, cardiovascular disease

## Abstract

**Background:** Mendelian randomization studies have so far restricted attention to linear associations relating the genetic instrument to the exposure, and the exposure to the outcome. In some cases, however, observational data suggest a non-linear association between exposure and outcome. For example, alcohol consumption is consistently reported as having a U-shaped association with cardiovascular events. In principle, Mendelian randomization could address concerns that the apparent protective effect of light-to-moderate drinking might reflect ‘sick-quitters’ and confounding.

**Methods:** The Alcohol-*ADH1B* Consortium was established to study the causal effects of alcohol consumption on cardiovascular events and biomarkers, using the single nucleotide polymorphism *rs1229984* in *ADH1B* as a genetic instrument. To assess non-linear causal effects in this study, we propose a novel method based on estimating local average treatment effects for discrete levels of the exposure range, then testing for a linear trend in those effects. Our method requires an assumption that the instrument has the same effect on exposure in all individuals. We conduct simulations examining the robustness of the method to violations of this assumption, and apply the method to the Alcohol-*ADH1B* Consortium data.

**Results:** Our method gave a conservative test for non-linearity under realistic violations of the key assumption. We found evidence for a non-linear causal effect of alcohol intake on several cardiovascular traits.

**Conclusions:** We believe our method is useful for inferring departure from linearity when only a binary instrument is available. We estimated non-linear causal effects of alcohol intake which could not have been estimated through standard instrumental variable approaches.

**Key Messages**

- Mendelian randomization studies have so far restricted attention to linear associations relating the genetic instrument to the exposure, and the exposure to the outcome, but this may not always be appropriate. For example, alcohol consumption is consistently reported as having a U-shaped association with cardiovascular events in observational studies.
- We propose a novel Mendelian randomization method based on estimating local average treatment effects for discrete levels of the exposure range, then testing for a linear trend in those effects.
- Our method gave a conservative test for non-linearity under realistic violations of the key assumption in simulations, and we believe our method is useful for inferring departure from linearity when only a binary instrument is available.
- We found evidence for a non-linear causal effect of alcohol intake on several cardiovascular traits in the Alcohol-*ADH1B* Consortium, using the single nucleotide polymorphism *rs1229984* in *ADH1B* as a genetic instrument.

## Introduction

Recent years have seen an increasing number of Mendelian randomization (MR) analyses that examine causal relationships between heritable exposures, such as levels of circulating biomarkers, and outcomes such as multifactorial diseases, for example coronary heart disease and type 2 diabetes.^1^^,^^2^^,^^3^ In principle, MR reduces problems of confounding and abolishes reverse causation by using a genetic proxy for the exposure in an instrumental variable (IV) analysis.^4^

To date, applications of MR have been limited to linear (or log-linear) models for the associations between gene and exposure and between exposure and outcome. In part this is because linear models have a natural interpretation which may be useful even if the true relationship is non-linear.^5^ Furthermore, many of the associations between genetic variants and complex traits discovered to date have appeared to be linear.^6^ However, in learning about causal relationships it is clearly of value to identify and characterize non-linear effects when they are present, bearing in mind that the existence and extent of such relationships may depend on the measurement scale. In particular, non-linear associations may translate into opposing effects (protective as well as harmful) according to the level of the exposure. Such opposing effects have been observed in many observational studies examining the relationship between alcohol consumption and cardiovascular events.^7^ Specifically, light-to-moderate levels of alcohol consumption have been associated with decreased risk of cardiovascular events relative to non-drinkers, with increased risk only occurring at higher levels of consumption. This apparent protective effect of light-to-moderate alcohol consumption could be explained by several different mechanisms, and corresponding ‘J’- or ‘U’-shaped associations have been observed with cardiovascular risk factors including low-density lipoprotein particles,^8^ abdominal adiposity,^9^ C-reactive protein (CRP),^10^^,^^11^ and triglycerides (TG).^12^ Similar observational associations were seen in our earlier analyses of *ADH1B* Consortium data (Holmes *et al*., Supplementary Appendix, Figure S3^13^).

As these observational findings suggest that light-to-moderate consumption may be cardio-protective, it is of great interest to consumers, suppliers and policy makers to establish whether this pattern is causal. Confounding is plausible, since socioeconomic groups that drink moderately may have other lifestyle factors that directly lead to lower rates of disease,^14^ and the relationship between confounders and alcohol may themselves be non-linear. Evidence for reverse causation is also well established, with those developing ill health or commencing medication more likely to reduce or quit alcohol consumption (the ‘sick-quitters’ phenomenon).^15^^,^^16^

Alcohol consumption is influenced by genetic variants that affect alcohol metabolism. Heritability of alcoholism has been estimated at 40–60%, and variants in *ALDH2*, *ADH1B* and *ADH1C* that encode for liver enzymes have been associated with decreased intake, via increased metabolism of alcohol to acetaldehyde or decreased acetaldehyde clearance, both leading to unpleasant side effects.^17^ In particular, *ADH1B* has been shown to be robustly associated with alcohol consumption^18^^,^^19^ and has been used in MR analyses to explore the causal effect of alcohol consumption on coronary heart disease risk factors.^20^

We recently established a large consortium (the ‘Alcohol-*ADH1B* Consortium’) of genetic association studies of European descent that used a single nucleotide polymorphism (SNP) in *ADH1B*, *rs1229984*, as the instrument to assess the impact of alcohol consumption on cardiovascular events and risk factors.^13^ This consortium showed that carrying the *rs1229984* A-allele was associated with non-drinking, lower alcohol consumption and lower incidence of binge drinking, which expands the previous associations of this variant with alcohol traits.^13^ Using a genetic association analysis, the consortium also showed that *ADH1B* carriers had a more favourable cardiovascular profile and a reduced risk of coronary heart disease (CHD).^13^ However, because of the existing literature on non-linear effects of alcohol consumption on cardiovascular events and the lack of appropriate methods to account for non-linear associations within IV analyses, we did not initially conduct an MR analysis in the Alcohol-*ADH1B* Consortium.

Approaches have been proposed for non-linear IV analysis in the econometric literature,^21–^^23^ but they cannot be used in this context because we use a single SNP as the IV. In the present paper, we develop new methods to conduct non-linear IV analysis using a single binary instrument, and also evaluate the impact of the key assumption of our method. We then apply our method to the data from the Alcohol-*ADH1B*Consortium to assess whether the causal effect of alcohol on cardiovascular traits is indeed non-linear and whether this implies a non-zero optimal level of consumption for cardiovascular health, which has clear implications for public health.

## Material and methods

### Data

The Alcohol-*ADH1B* Consortium is a collaboration of studies in which the associations between an allele of the *ADH1B* gene and 22 cardiovascular biomarkers and risks of coronary heart disease, stroke and type 2 diabetes have been examined.^13^ Here our analyses are restricted to the 22 studies (18 cohorts, 2 nested case-control studies, 1 randomized trial and1 case-control study) with individual participant data originating from Europe (*n* = 16) and North America (*n* = 6). Analysis was restricted to individuals of European descent.^13^

The principal alcohol trait was weekly volume of alcohol in British units [1 British unit is equivalent to 0.57 US units or 10 ml (7.9 g) ethanol], which we derived using questionnaire data from each study. For studies in which this variable was not already present, we either calculated weekly volume of alcohol by summing over the individual components of beverage-specific drink questions (available in 20 of the 22 studies), or by converting alcohol recorded in g/week into British units.^13^ The units/week were log-transformed, after incrementing by one to allow for individuals reporting zero weekly alcohol consumption, resulting in a normally distributed phenotype that had homoscedastic residual error after regressing on the *ADH1B* genotype.

Here we considered a subset of outcomes for which a non-linear causal association was either postulated from subject-matter knowledge, or suggested by the observational data available from the Alcohol-*ADH1B* Consortium (all *P* < 0.001 for the quadratic term in a quadratic model): systolic blood pressure (SBP), non high-density lipoprotein cholesterol (non-HDL-C),TG, high-density lipoprotein cholesterol (HDL-C), body mass index (BMI), waist circumference (WC), CRP and interleukin 6 (IL-6). Outcomes were log-transformed towards normality when appropriate (TG, CRP and IL-6).

The *rs1229984* polymorphism in *ADH1B* was directly genotyped in all studies and coded as 0/1 according to the carriage of at least one minor allele. This coding was adopted owing both to the low prevalence of the *rs1229984*A-allele (average carriage of *rs1229984*A-alleles in the analysis sample: 7.7%) and the stronger association observed with alcohol dependence and other alcohol-related traits under a dominant model compared with a recessive model.^24^

Full details of participating studies, phenotype definition and genotyping are reported elsewhere^13^ and are summarized in Table S1 in the Supplementary data, available at *IJE* online.

### Linear instrumental variable analysis

We used standard two-stage least squares (2SLS) to estimate a linear causal effect of log(weekly units of alcohol+1) (hereafter, log-alcohol) on continuous cardiovascular outcomes. That is, we fitted the first-stage linear regression

$$x_{i}=\beta_{XG}g_{i}+\beta_{XZ}^{\prime }z_{i}+\varepsilon_{Xi}$$

where $x_{i}$ is log-alcohol for subject i, $g_{i}$ is a binary code for the *rs1229984* genotype, $z_{i}$ is a vector of covariates and $\varepsilon_{Xi}$ are residual errors assumed to be independent and identically distributed with mean zero. Regression coefficients $\beta_{XG}$ and $\beta_{XZ}$ were estimated as fixed effects. We used the fitted model to predict ${\hat{x}}_{i}$ then estimated the alcohol-outcome association $\beta_{YX}$ from the regression

$$y_{i}=\beta_{YX}{\hat{x}}_{i}+\beta_{YZ}^{\prime }z_{i}+\varepsilon_{Yi}$$

where $y_{i}$ is the continuous cardiovascular outcome for subject i and $\varepsilon_{Yi}$ are residual errors assumed to be independent and identically distributed with mean zero. A 95% confidence interval (CI) for ${\hat{\beta }}_{YX}$ was derived by nesting the 2SLS within a bootstrap resampling procedure using 10 000 bootstrap samples. As covariates we included in both regressions a fixed effect for each study and fixed effects for age and sex.

### Non-linear causal effects

**Figure 1.**
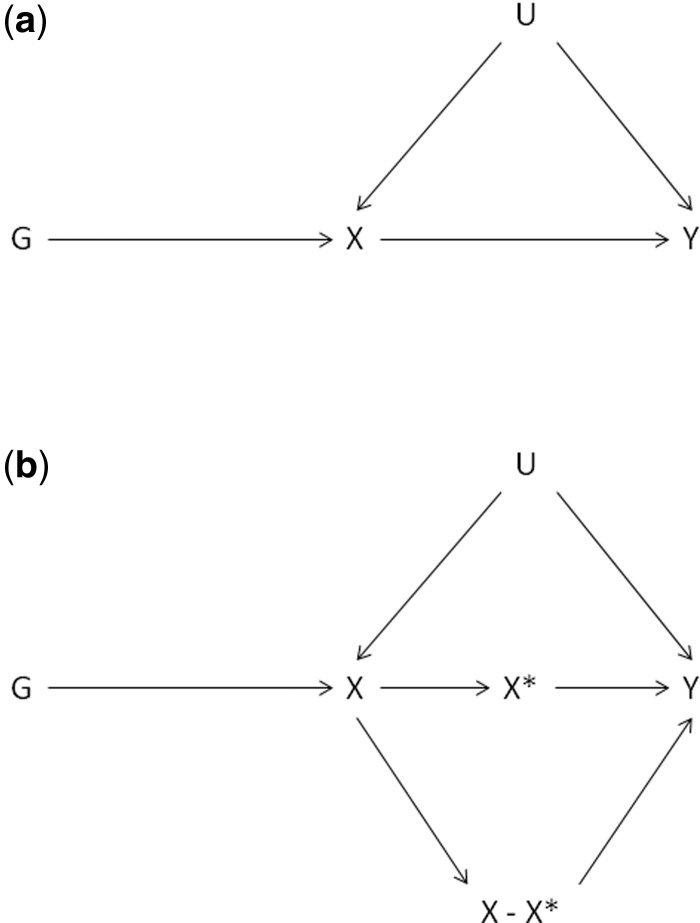
Directed acyclic graphs encoding a) the standard Mendelian randomization assumptions: (i) G is associated with X, (ii) G is not associated with confounders U of the X-Y association, and (iii) G affects Y only via its association with X; (b) how these assumptions are affected by the discretization of X in the proposed non-linear Mendelian randomization approach.

To test for non-linearity of the causal *X-Y* association we consider local average treatment effects (LATEs) in subgroups of *X*.^25^ First we coarsen *X* into a discrete and rescaled variable $X^{*}=\left\lfloor \frac{X}{\beta_{XG}}\right\rfloor$ with finite support, assumed without loss of generality to be {0,…,J} for fixed *J*. *G* is an instrument for $X^{*}$ if it is independent of the remainder $X-X^{*}$ (see Figure 1); this is not generally true but it can be tested in applications. Under linear models we can obtain an estimate of the causal effect of $X^{*}$ on Y, but this effect can also be represented as a weighted sum of LATEs,^25^^,^^26^ which are causal effects among the individuals whose exposures $X^{*}$ are changed from one level to the next by the genetic instrument.

More precisely, let $Y_{i}(j)$ denote the potential outcome for subject *i* obtained by setting, possibly contrary to fact, the exposure $X_{i}^{*}=j$. Moreover let $X_{i}^{*}(0)$ and $X_{i}^{*}(1)$ be the possibly counterfactual values of the exposure obtained by setting the binary instrument to 0 and 1 respectively. Then the LATE at exposure level *j* is defined as

$$\tau_{j}=E[Y_{i}(j)-Y_{i}(j-1)|X_{i}^{*}(1)\geq j>X_{i}^{*}(0)]$$

that is, the average treatment effect among those whose exposure would be at least *j* if their instrument were set to 1, and whose exposure would be less than *j* if their instrument were set to 0. Identification of LATEs requires the further assumption of monotonicity, that is either $X_{i}^{*}(1)-X_{i}^{*}(0)\geq 0$ or $X_{i}^{*}(1)-X_{i}^{*}(0)\leq 0$ for all subjects *i*, implying that the instrument either does not decrease the exposure in all subjects, or does not increase it in all subjects.

If we could estimate the LATEs $\tau_{j}$ then testing them for equality would provide a direct test of linearity of the causal effect. Here we propose an assumption that allows this to be performed. Assume that the causal effect of the instrument on the discretized exposure is exactly 1 in each subject:

$$X_{i}^{*}(1)-X_{i}^{*}(0)=1\forall i\text{.}$$

This is a stronger version of the monotonicity assumption. In fact, this assumption will hold if the first-stage linear model is a true structural model for *X*, with no unmeasured confounders of the *G-X* association, or modifiers of the effect of *G* on *X*. Under this assumption (and noting that X has been rescaled so that a one unit change in $X^{*}$ corresponds to the expected exposure change with genotype), every subject contributes to a LATE, since for every *i* there is a *j* such that $X_{i}^{*}(1)\geq j>X_{i}^{*}(0)$, in fact $X_{i}^{*}(1)=j=X_{i}^{*}(0)+1$. That is, the instrument moves each subject from one level of $X^{*}$ to the next: in the randomized trials terminology, all subjects are compliers.

It is now possible to assign each subject to the estimation of a LATE, based on the observed data. Since $X_{i}^{*}(1)=j=X_{i}^{*}(0)+1$ if and only if $X_{i}^{*}=j$ and $G_{i}=1$ or $X_{i}^{*}=j-1$ and $G_{i}=0$, we can write the LATE as

$$\begin{array}{c} \tau_{j}=E[Y_{i}(j)-Y_{i}(j-1)|X_{i}^{*}(1)\geq j>X_{i}^{*}(0)]\\ =E[Y_{i}(j)|X_{i}^{*}=j,G_{i}=1\vee X_{i}^{*}=j-1,G_{i}=0]\\ -E[Y_{i}(j-1)|X_{i}^{*}=j,G_{i}=1\vee X_{i}^{*}=j-1,G_{i}=0]\\ =E[Y|X_{i}^{*}=j,G_{i}=1]-E[Y|X_{i}^{*}=j-1,G_{i}=0] \end{array}$$

which may be estimated using ordinary linear regression (possibly with adjustment for relevant covariates) restricted to the subjects having $X_{i}^{*}=j$ and $G_{i}=1$ or $X_{i}^{*}=j-1$ and $G_{i}=0$.

Having estimated a LATE (with its standard error) for each level of $X^{*}$, the estimates may be tested for equality using standard methods of meta-analysis. In particular, we use meta-regression to test for a linear trend in the LATEs. A linear model relating LATEs to the exposure levels

$$E(\tau_{j})=\gamma_{1}+\gamma_{2}j$$

would apply if the underlying causal model were quadratic

$$E(Y)=\gamma_{0}+\gamma_{1}j+\frac{1}{2}\gamma_{2}j^{2}\text{.}$$

The coefficient $\gamma_{2}$ is zero if the LATEs are equal, which is the case when the causal effect of *X* on *Y* is linear. Then the mean LATE, calculated by fixed-effects meta-analysis of the estimated LATEs, is an alternative measure of the linear causal effect of *X*. Rejection of $\gamma_{2}=0$ implies a non-linear causal effect; a quadratic form is not directly implied but such a model could be hypothesized, up to its intercept term, from the fitted meta-regression. The estimation of a linear model relating LATEs to the exposure levels is a simple but powerful way to investigate departures from linearity, as any such departures are captured by a single parameter. However, alternative models could be fitted to characterize the dose-response relationship more flexibly. For example, a piecewise constant model relating the LATEs to the exposure levels would correspond to a linear spline model relating the exposure to the outcome. This could be detected by a test of Cochran’s Q on the estimated LATEs.

This procedure requires rescaling of *X* by the effect size $\beta_{XG}$ of the instrument. However the true value of $\beta_{XG}$ is unknown and it must be estimated. To account for sampling uncertainty in ${\hat{\beta }}_{XG}$ we nest the entire LATE and meta-regression procedure within a bootstrap resampling procedure, using 10 000 bootstrap samples, to obtain proper confidence intervals on the meta-regression estimates ${\hat{\gamma }}_{1},{\hat{\gamma }}_{2}$. Our procedure for testing departure from linearity of the causal effect of *X* on *Y* is summarized in Box 1.

**Box 1.Summary of proposed method for testing for a non-linear causal effect**

1. For the observed data and for each of *K* bootstrap samples:
  1.1 Regress *X* on *G* for all subjects, giving estimated regression coefficient ${\hat{\beta }}_{XG}$
  1.2 Discretize *X* into units of ${\hat{\beta }}_{XG}$, that is derive the discrete variable $X^{*}=\;\left\lfloor \frac{X}{{\hat{\beta }}_{XG}}\right\rfloor$
  1.3 For each discrete value of *j*:
    1.3.1 Regress *Y* on $X^{*}$ using only the subjects for which $X_{i}^{*}=j$ and $G_{i}=1$, or $X_{i}^{*\prime }=j-1$ and $G_{i}=0$. Among these subjects there is no variation in $X^{*}$ that is not explained by *G*.
    1.3.2 This yields ${\hat{\tau }}_{j}$, the estimated local average treatment effect (LATE) for level *j* of $X^{*}$
    1.3.3 Rescale ${\hat{\tau }}_{j}$ by ${\hat{\beta }}_{XG}$ to the original scale of *X*
  1.4 Obtain the mean LATE by fixed-effects meta-analysis of ${\hat{\tau }}_{j}$
  1.5 Meta-regress ${\hat{\tau }}_{j}$ on *j* to obtain the intercept and slope of the LATEs, corresponding to a quadratic causal model.
2. Obtain empirical confidence intervals on the mean LATE and the LATE intercept and slope from the bootstrap samples.

Beyond a test for departure from linearity, we are interested in identifying the way the causal effect changes with increasing alcohol consumption and, in particular, the nadir of the curve which could be conceived as an ‘optimal’ level of consumption regarding cardiovascular traits. As we cannot estimate the intercept term in the fitted quadratic model, we cannot predict the absolute value of the outcome for a given level of alcohol consumption, so we focus on the difference in outcome relative to zero alcohol consumption. For those outcomes with evidence of non-linearity, we predict this at four values of alcohol consumption (3.04, 12.15, 31.90 and 84.52 units/week), which are the medians of observed values in the categories representing low (>0–7 units/week), moderate (7–21 units/week), heavy (21–70 units/week) and very heavy (70+ units/week) alcohol consumption in the analysis of Holmes *et al*.^13^ By differentiation of the hypothesized quadratic function, we estimate three additional features of the curve: (i) the ‘optimal’ level of alcohol consumption; (ii) the difference in outcome at the optimal alcohol consumption relative to zero alcohol consumption; and (iii) the level of alcohol consumption required to have an outcome level equivalent to that at zero alcohol consumption. Confidence intervals for all the estimates are obtained by nesting the estimation within the bootstrap resampling procedure outlined above. In the bootstrap samples we left truncated the nadir of alcohol consumption at zero.

All analyses were conducted using R version 2.13.^27^

### Simulations

We conducted simulations to assess the proposed approach in terms of bias and coverage under various data-generating models. Full details and results are given in the Supplementary data, available at *IJE* online. In brief, we simulated data in which there was no causal *X-Y* association, in which the association was linear and in which there was a quadratic causal association, allowing throughout for quadratic effects of confounders. We assessed robustness to the assumption of individual-level homogeneity of the genetic effect using additional simulations of $\beta_{XG}$ heterogeneity and *G-U* interaction at both the individual and subgroup levels.

We observed that the LATE estimates were essentially unbiased with generally good coverage properties under null, linear and quadratic models, and that the test for a non-linear effect was slightly conservative. Together the results suggest that this method is a useful extension to standard approaches in the non-linear setting. Reasonable levels of individual-level heterogeneity in $\beta_{XG}$ or between-subgroup heterogeneity in $\beta_{XG}$ were not found to lead to significant bias in the estimates. High levels of interaction between G and U led to bias in the estimates, but such interactions may be unlikely in practice.

## Results

We investigated the potential non-linear effects of log-alcohol on each of the outcomes in the Alcohol-*ADH1B* Consortium using the proposed procedure. Some issues relating to the inclusion of multiple studies in the Consortium are discussed in the Supplementary data, available at *IJE* online.

Age- and sex-adjusted study-specific estimates of the association between *rs1229984* and log-alcohol are presented in Figure S17 of the Supplementary data, available at *IJE* online. These study-specific estimates have (inverse-variance-weighted) mean −0.235 and standard deviation (SD) 0.121, indicating some degree of between-study variability. However, in our simulations (see Supplementary data, available at *IJE* online) a similar degree of heterogeneity between known subgroups (scenario ‘f’ with γ=0.1) was not found to result in bias to either the LATE intercept or slope, with slightly conservative confidence intervals for each.

To examine whether *G* = *rs1229984* is a valid instrument for discretized $X^{*}$, assuming that it is valid for the continuous measure X = log-alcohol, we examined the correlation between *G* and the remainder $X-X^{*}$; these should be independent for *G* to be a valid instrument for $X^{*}$. We observed a weak but significant correlation (Pearson’s *r* = −0.013, 95% CI: −0.020, −0.006). We hypothesized that this residual correlation was due to the large number of individuals reporting drinking zero weekly units of alcohol (log-alcohol = 0), because these individuals have a residual $X-X^{*}$ = 0 and are also more likely to have *G* = 0. When individuals with log-alcohol = 0 were excluded from the analysis, the correlation between *G* and the remainder $X-X^{*}$ was close to zero (Pearson’s *r* = 0.001, 95% CI: −0.007, 0.009). We therefore re-analysed the data after excluding individuals with log-alcohol = 0, but obtained very similar results to those from the full sample. Because it is necessary to retain individuals reporting zero drinking to meet the objectives of the analysis, we only report results using the full sample.

The results of the LATE-based analysis for each of the outcomes are presented in Table 1 along with the standard linear IV analysis. We illustrate our approach in more detail using SBP as an example, following the steps in Box 1. We estimated ${\hat{\beta }}_{XG}=-0.244$ assuming a common genetic effect across all studies. Discretizing log-alcohol into units of −0.244 gave an integer exposure $X^{*}$ with range [−26,0]. We then estimated the LATE at each value of $X^{*}$. For example, for j=−11 [corresponding to a log-alcohol of −11 × −0.244 = 2.684, or exp(2.684) – 1 = 13.6 units/week] we selected the subjects with $X^{*}=-11$ and *rs1229984* = 1, or $X^{*}=-12$ and *rs1229984* = 0. Linear regression of SBP on $X^{*}$, on these subjects only, and adjusting for study, age and sex, gave $\tau_{-11}=-1.55$; that is, in subjects whose $X^{*}$ was changed from −12 to −11 by the SNP, their SBP was decreased by 1.55 mmHg.

**Table 1.** Comparison of linear and non-linear instrumental variable estimates for selected cardiovascular traits in the Alcohol-*ADH1B* Consortium

Outcome	*n*	Linear IV approach		Non-linear IV approach						
				Mean LATE		LATE intercept		LATE slope		
		Estimate	95% CI^a^	Estimate	95% CI^a^	Estimate	95% CI^a^	Estimate	95% CI^a^	*P*^b^
SBP (mmHg)	78172	5.20	3.2, 7.3	4.90	2.6, 7.5	−2.20	−7.5, 3.4	3.30	1.0, 5.5	0.004
Non-HDL-C (mmol/l)	60140	0.13	−0.02, 0.28	0.25	0.06, 0.45	−0.54	−0.94, −0.120	0.37	0.19, 0.55	<0.001
HDL-C (mmol/l)	60227	−0.02	−0.07, 0.03	−0.01	−0.07, 0.06	−0.02	−0.15, 0.14	0.00	−0.06, 0.06	0.910
BMI (kg/m^2^)	79454	0.70	0.2, 1.2	1.00	0.4, 1.5	−1.00	−2.5, 0.3	0.90	0.3, 1.4	0.002
WC (cm)	57172	2.80	1.3, 4.4	2.70	1.1, 4.5	−1.80	−5.8, 1.9	2.00	0.6, 3.6	0.010
CRP^c^ (mg/l)	63367	0.17	0.03, 0.31	0.18	0.03, 0.38	−0.39	−0.77, 0.03	0.26	0.10, 0.43	0.001
IL-6^c^ (pg/ml)	23535	0.30	0.16, 0.45	0.35	0.10, 0.53	0.10	−0.24, 0.85	0.13	−0.34, 0.29	0.410
TG^c^ (mmol/l)	63667	0.01	−0.06, 0.07	0.01	−0.09, 0.07	0.04	−0.15, 0.21	−0.02	−0.10, 0.06	0.670

SBP, systolic blood pressure; Non-HDL-C, non high-density lipoprotein cholesterol; HDL-C, high-density lipoprotein cholesterol; BMI, body mass index; WC, waist circumference; CRP, C-reactive protein; IL-6, interleukin 6; TG, triglycerides.

^a^Derived using 10 000 bootstrap samples.

^b^Approximate Z-test using the bootstrap standard error.

^c^Log-transformed prior to analysis.

Rescaling by ${\hat{\beta }}_{XG}=-0.244$, subjects whose log-alcohol was changed from −12×−0.244=2.928 to −11×−0.244=2.684 [i.e. whose weekly units of alcohol consumption was changed from exp(2.928) – 1 = 17.7 to exp(2.684) – 1 = 13.6] by the SNP had their SBP decreased by 1.55 mmHg. Alternatively, a one-unit increase in log-alcohol at this level of alcohol consumption [e.g. from 2.684 to 3.684, or from exp(2.684) – 1 = 13.6 to exp(3.684) – 1 = 38.8 units/week—a considerable increase] was associated with an increase in SBP of −1.55/−0.244=6.35 mmHg.

**Figure 2.**
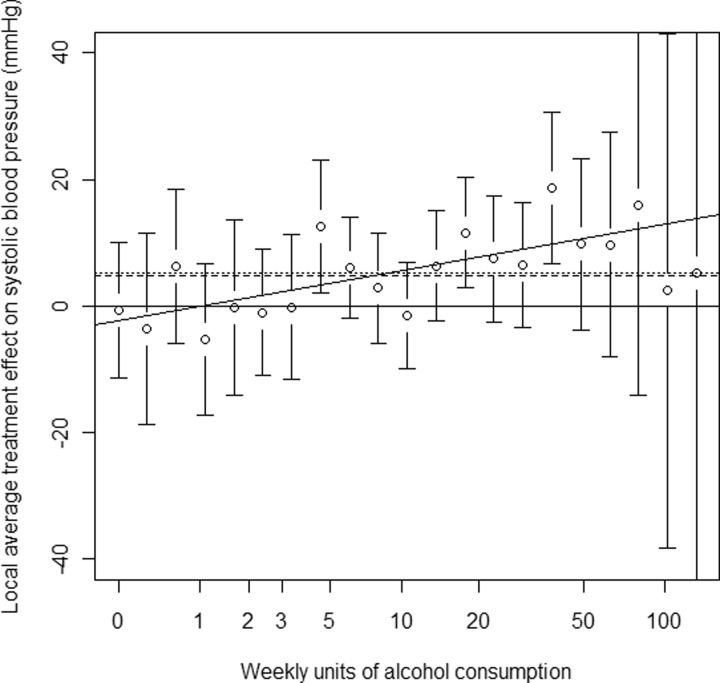
Local average treatment effects (LATEs) of log(weekly units of alcohol + 1) on systolic blood pressure. Circular markers are LATEs; bars are 95% pointwise confidence intervals; dashed line is estimated mean LATE; solid line is estimated linear LATE trend; dotted line is linear IV estimate using the ratio method (virtually indistinguishable from the estimated mean LATE).

The full graph of estimated LATEs for SBP is shown in Figure 2. Negative LATEs represent decreasing SBP with log-alcohol whereas positive LATEs represent increasing SBP, so a LATE trend crossing zero from negative to positive indicates a nadir. Fixed-effects meta-analysis of these effects gave a mean LATE of 4.9 (95% CI: 2.6, 7.5), which is effectively a complier average treatment effect and similar to the linear IV estimate of 5.2 (95% CI: 3.2, 7.3). Meta-regression of the estimated LATEs on $X^{*}$ gave a slope of 3.3 (95% CI: 1.0, 5.5). This provided strong evidence (Z-test *P* = 0.004) that the LATEs were not constant across values of log-alcohol; that is, there was a non-linear association between log-alcohol and SBP.

Full results for the remaining outcomes are provided in Table 1. As indicated by the LATE slope, there was evidence of a non-linear causal effect for SBP, non-HDL-C, BMI, WC and CRP (all *P* ≤ 0.01). For other outcomes there was no evidence of a non-linear causal effect (HDL-C, IL-6 and triglycerides, all *P* > 0.4, though note that power is lower for IL-6 due to the relatively small sample size). In these cases we recommend that the linear IV results are employed, as fewer assumptions are required in their estimation. It should also be noted that the linear IV estimates and the mean LATEs were similar for each of the outcomes, albeit with the latter having wider CIs.

Table 2 shows the predicted difference in each outcome relative to zero alcohol consumption for 3.04, 12.15, 31.90 and 84.52 units/week of alcohol consumption under the fitted quadratic functions. All outcomes, with the exception of SBP, were predicted to be lower at 3.04 units/week (‘low’ alcohol consumption) than at zero alcohol consumption, though each confidence interval included the possibility of no true difference. By 31.90 units/week (‘heavy’ alcohol consumption) all outcomes were predicted to be higher than at zero alcohol consumption, though each confidence interval, with the exception of SBP, again included the possibility of no true difference. By 84.52 units/week (‘very heavy’ alcohol consumption) all the confidence intervals excluded the possibility of no true difference.

**Table 2.** Predicted difference in cardiovascular traits relative to zero alcohol consumption at several levels of alcohol consumption and predicted curve features in the Alcohol-*ADH1B* Consortium. Only calculated for traits with evidence of non-linearity

Outcome	Difference in outcome (95% CI^a^)				Level of alcohol consumption at nadir (units/week) (95% CI^a^)	Difference in outcome at optimal alcohol consumption (95% CI^a^)	Level of alcohol consumption with outcome equal to that at zero (units/week) (95% CI^a^)
	3.04 units/week^c^	12.15 units/week^c^	31.90 units/week^c^	84.52 units/week^c^			
SBP (mmHg)	0.1 (−5.5, 6.1)	5.2 (−2.6, 13.9)	12.4 (3.4, 22.1)	22.8 (12.2, 34.6)	1.0 (0.0, 3.6)	−0.7 (−5.4, 0.0)	2.8 (0.0, 19.6)
Non-HDL-C (mmol/l)	−0.39 (−0.79, 0.06)	−0.15 (−0.72, 0.47)	0.40 (−0.28, 1.10)	1.30 (0.45, 2.16)	3.2 (0.7, 6.0)	−0.39 (−0.85, −0.03)	16.9 (2.1, 48.2)
BMI (kg/m^2^)	−0.6 (−2.2, 0.8)	0.2 (−2.0, 2.1)	1.6 (−0.8, 3.8)	3.9 (1.2, 6.3)	2.3 (0.0, 6.0)	−0.6 (−2.3, 0.0)	10.1 (0.0, 48.4)
WC (cm)	−0.6 (−4.7, 3.5)	1.9 (−3.9, 7.8)	5.7 (−0.6, 12.5)	11.5 (4.5, 19.2)	1.5 (0.0, 5.4)	−0.8 (−4.9, 0.0)	5.3 (0.0, 37.4)
CRP^b^ (mg/l)	−0.29 (−0.68, 0.15)	−0.15 (−0.68, 0.50)	0.22 (−0.37, 0.95)	0.83 (0.15, 1.69)	3.5 (0.0, 7.2)	−0.30 (−0.75, 0.00)	19.4 (0.0, 66.0)

SBP, systolic blood pressure; Non-HDL-C, non high-density lipoprotein cholesterol; BMI, body mass index; WC, waist circumference; CRP, C-reactive protein.

^a^Derived using 10 000 bootstrap samples.

^b^Log-transformed prior to analysis.

^c^Weekly units of alcohol values are medians of observed values in categories representing low (1–7 units/week), moderate (7–21 units/week), heavy (21–70 units/week) and very heavy (70+ units/week) alcohol consumption in the analysis of Holmes *et al.*^13^

**Figure 3.**
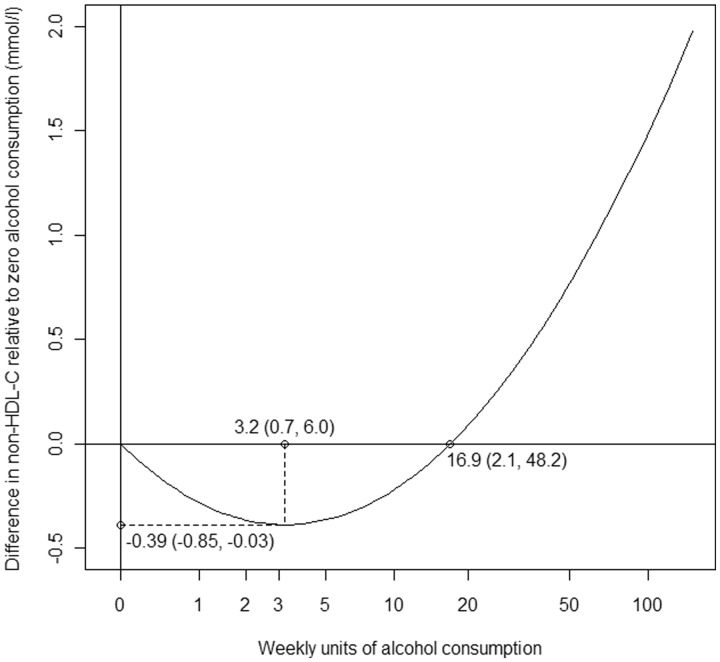
Predicted difference in non high-density lipoprotein cholesterol (non-HDL-C) relative to zero alcohol consumption across the range of values of observed alcohol consumption, with estimated optimal level of alcohol consumption (3.2 (95% confidence interval (CI): 0.7, 6.0) units/week), estimated difference in non-HDL-C relative to zero alcohol consumption at optimal level (−0.39 (95% CI: −0.85, −0.03) mmol/l), and estimated level of alcohol consumption with the same level of non-HDL-C as at zero (16.9 (95% CI: 2.1, 48.2) units/week) indicated.

Table 2 also shows the additional estimated features of the hypothesized quadratic functions. For all outcomes, the optimal level of alcohol consumption was estimated to be greater than zero, ranging from 1.0 units/week (SBP) to 3.5 units/week (CRP). However, only for non-HDL-C did the confidence interval exclude the possibility that zero consumption may be optimal. Correspondingly, the estimated difference in outcome at the optimal alcohol consumption level relative to zero consumption was negative for each outcome, though only for non-HDL-C did the confidence interval exclude the possibility of no true difference. The level of alcohol consumption required to have an outcome level equivalent to that at zero consumption was estimated as ranging from 2.8 units/week (SBP) to 19.4 units/week (CRP), though for all outcomes the confidence intervals were very wide. These results are illustrated for non-HDL-C, for which the strongest evidence of non-linearity was observed, in Figure 3. However, the precise values of our quantitative results should be interpreted with some caution as the quadratic causal model that we fit may not be sufficiently flexible to fully characterize the dose-response relationship.

## Discussion

We have proposed a method based on estimating LATEs that allows a basic estimation of local causal effects of a continuous exposure when using a binary instrument. Our method requires an assumption of homogeneous individual treatment effects of the instrument on the exposure, but our simulations found the estimates obtained under our approach to be largely unbiased and with good coverage properties under a variety of heterogeneous effects of instrument on exposure.

The local effects we estimate are within discretized units of the exposure, with the size of those units depending on the gene-exposure association. This is not a scale with a generally useful interpretation, and different genetic instruments could lead to different discrete units with different definitions of local causal effects. We therefore emphasize the ability to test for a non-linear causal effect and draw qualitative conclusions about the shape of that effect, and we suggest that a strictly quantitative interpretation of the estimated parameters should be viewed with some caution. Further work is required in investigating alternative models relating the LATEs to the exposure levels in order to provide greater flexibility for characterizing the dose-response relationship.

Using this approach, we detected evidence for a non-linear causal effect of log-alcohol on several cardiovascular traits in a large collaborative study, which would not have been possible using standard IV approaches. For each outcome that exhibited evidence of a non-linear causal effect, our results suggested that the level of alcohol consumption associated with the lowest value of the cardiovascular traits to lie between 1.0 and 3.5 units/week. However, only for non-HDL-C do we have strong evidence that the optimal level of consumption truly differs from zero.

As the cardiovascular traits considered in this analysis were observed concurrently with the level of alcohol consumption in many of the studies within the *ADH1B* Consortium, a conventional analysis would be at risk of bias due to reverse causality (for example, someone with high SBP reducing their alcohol intake so that they are observed to have a low level of consumption). A Mendelian randomization analysis removes the possibility of such reverse causality, which is a significant strength of the present study.

For our estimated effects to be interpreted causally we need the standard assumptions underlying MR analysis to hold. Of particular concern in the present application is the exclusion restriction that *G* has no effect on *Y* other than through *X*. We have only considered one aspect of alcohol consumption (weekly units), but if the polymorphism in *ADH1B* reduces alcohol consumption generally, then other aspects, such as frequency of binge drinking, may also be associated with the instrument.^19^ If such other aspects have a causal effect on the outcome independently of weekly units, then the exclusion restriction would not hold. The strong correlation between weekly units and other aspects of alcohol consumption makes a significant violation of this assumption unlikely. However, further research is required in this area.

Although we limited our analyses to individuals of European descent and adjusted for study in all our analyses, there may be residual population stratification of the variant which could lead to backdoor pathways from the instrument to the outcome. The restriction to individuals of European descent may also reduce the generalizability of our findings beyond such populations.

An inherent aspect of our approach is the need for a large sample with a sufficiently strong association between the gene and the exposure. If the gene-exposure association is very weak, then the exposure will be discretized into many bins, none of which will contain sufficient subjects for the LATEs to be estimated. Many MR studies are now conducted on large samples in order to improve power to detect causal effects, but our approach requires large samples across a sufficient range of the exposure in order to detect non-linearities. This problem is compounded when studying binary outcomes, as each bin should contain a sufficient number of events. Therefore we have restricted our attention to continuous outcomes in this paper, but we recognize that here the key interest is in the nature of the causal relationship with cardiovascular disease events, which cannot be readily deduced from the associations with different risk factors. Further work in this area is required.

We believe our method is useful for inferring departure from linearity when only a binary instrument is available. Although there is clearly greater scope for bias than in standard IV analysis, we did not infer non-linear effects for several of the cardiovascular outcomes we considered, suggesting some degree of specificity using our method. More robust inference of non-linear causal effects may be possible from polychotomous or continuous instruments, such as gene scores constructed from multiple SNPs.^28^^,^^29^ Such instruments will allow the identification of non-linear models with many parameters, though IV estimation of parametric non-linear models has been found to be dependent on the choice of parametric model.^23^ A further key issue is whether the exposures predicted by those instruments cover a sufficient range to capture the non-linear features of the causal effects. If this is not the case, then it may be necessary to pursue approaches based on local effects, similar to the one for binary instruments that we have discussed here.

## Supplementary Data

Supplementary data are available at *IJE* online.

## Funding

This work was supported by the UK Economic and Social Research Council (NCRM Pathways node ES/I025561/2 to R.S.) and the UK Medical Research Council (Population Health Scientist Fellowship G0802432 to M.V.H and G1000718 to F.D.). D.A.L. and G.D.S. work in a unit that receives funding from the UK Medical Research Council (MC_UU_12013/1–9) and the University of Bristol.

## Supplementary Material

Supplementary Data
