# Supplementary material for: Testing for non-linear causal effects using a binary genotype in a Mendelian randomization study: application to alcohol and cardiovascular traits
Source: Int J Epidemiol. 2014 Sep 5;43(6):1781–90. doi: 10.1093/ije/dyu187 (PMC4276061; doi:10.1093/ije/dyu187)
Supplement: Supplementary Data [file supp_43_6_1781__index.html]

Testing for non-linear causal effects using a binary genotype in a Mendelian randomization study: application to alcohol and cardiovascular traits — Testing for non-linear causal effects using a binary genotype in a Mendelian randomization study: application to alcohol and cardiovascular traits — Testing for non-linear causal effects using a binary genotype in a Mendelian randomization study: application to alcohol and cardiovascular traits — Supplementary Data 

# Testing for non-linear causal effects using a binary genotype in a Mendelian randomization study: application to alcohol and cardiovascular traits

## Supplementary Data

files

**Files in this Data Supplement:**

- Supplementary Data - docx file
- Supplementary Data - docx file
