# Supplementary material for: Testing for non-linear causal effects using a binary genotype in a Mendelian randomization study: application to alcohol and cardiovascular traits
Source: Int J Epidemiol. 2014 Sep 5;43(6):1781–90. doi: 10.1093/ije/dyu187 (PMC4276061; doi:10.1093/ije/dyu187)
Supplement: Supplementary Data [file supp_dyu187_dyu187Group_acknowledgements.docx]

Alcohol-*ADH1B* Consortium Acknowledgements

**ARIC**: The Atherosclerosis Risk in Communities Study is carried out as a collaborative study supported by National Heart, Lung, and Blood Institute contracts (HHSN268201100005C, HHSN268201100006C, HHSN268201100007C, HHSN268201100008C, HHSN268201100009C, HHSN268201100010C, HHSN268201100011C, and HHSN268201100012C), R01HL087641, R01HL59367 and R01HL086694; National Human Genome Research Institute contract U01HG004402; and National Institutes of Health contract HHSN268200625226C. The authors thank the staff and participants of the ARIC study for their important contributions. Infrastructure was partly supported by Grant Number UL1RR025005, a component of the National Institutes of Health and NIH Roadmap for Medical Research; **BRHS**: The British Regional Heart Study has been supported by programme grant funding from the British Heart Foundation (RG/08/013/25942); **BWHHS**: The British Women’s Heart and Health Study has been supported by funding from the British Heart Foundation (BHF) (grant PG/09/022) and the UK Department of Health Policy Research Programme (England) (grant 0090049). The BWHHS HumanCVD data were funded by the BHF (PG/07/131/24254).; We thank all BWHHS participants, the general practitioners and their staff who have supported data collection since the study inception; **CARDIA** is conducted and supported by the National Heart, Lung, and Blood Institute in collaboration with the University of Alabama at Birmingham (HHSN268201300025C & HHSN268201300026C), Northwestern University (HHSN268201300027C), University of Minnesota (HHSN268201300028C), Kaiser Foundation Research Institute (HHSN268201300029C), and Johns Hopkins University School of Medicine (HHSN268200900041C). CARDIA is also partially supported by the Intramural Research Program of the National Institute on Aging; **CHS**: This research was supported by contracts HHSN268201200036C, HHSN268200800007C, N01 HC55222, N01HC85079, N01HC85080, N01HC85081, N01HC85082, N01HC85083, N01HC85086, N01HC65226, and grant HL080295 from the National Heart, Lung, and Blood Institute (NHLBI), with additional contribution from the National Institute of Neurological Disorders and Stroke (NINDS). Additional support was provided by AG023629 from the National Institute on Aging (NIA). A full list of principal CHS investigators and institutions can be found at CHS-NHLBI.org; **Cyprus**: The Cyprus Study has been supported by the Cyprus Cardiovascular Disease Educational and Research Trust (CCDERT) and Joint Cyprus Research Promotion Foundation, Ministry of Health and Cyprus Heart Foundation grant No 41/5PE as well as Research Promotion Foundation grants (PENEK 05/04 and YGEIA 04/06); **Czech post-MONICA**: **Supported by MH CZ - DRO ("Institute for Clinical and Experimental Medicine - IKEM, IN 00023001"**). **EAS**: The EAS was funded by the British Heart Foundation (Programme Grant RG/98002); **ELSA**: Samples from the English Longitudinal Study of Ageing (ELSA) DNA Repository (EDNAR), received support under a grant (AG1764406S1) awarded by the National Institute on Ageing (NIA). ELSA was developed by a team of researchers based at the National Centre for Social Research, University College London and the Institute of Fiscal Studies. The data were collected by the National Centre for Social Research.; **EPIC Turin**: The EPIC Turin study is funded by grants from the Associazione Italiana per le Ricerche sul Cancro, Italy and grants from the Compagnia di San Paolo, Turin, Italy; **FHS**: The Framingham Heart Study began in 1948 with the recruitment of an original cohort of 5,209 men and women (mean age 44 years; 55 percent women). In 1971 a second generation of study participants was enrolled; this cohort consisted of 5,124 children and spouses of children of the original cohort. The mean age of the offspring cohort was 37 years; 52 percent were women. A third generation cohort of 4,095 children of offspring cohort participants (mean age 40 years; 53 percent women) was enrolled beginning in 2002. At each clinic visit, a medical history was obtained with a focus on cardiovascular content, and participants underwent a physical examination including measurement of height and weight from which BMI was calculated; **HAPIEE**: This study was supported by Wellcome Trust ‘Determinants of Cardiovascular Diseases in Eastern Europe: A multi-centre cohort study’ [grants 064947/Z/01/Z; and 081081/Z/06/Z]; the MacArthur Foundation ‘MacArthur Initiative on Social Upheaval and Health’ [grant 712058]; the National Institute on Ageing ‘Health disparities and aging in societies in transition (the HAPIEE study)’ [grant 1R01 AG23522]; and a project from the Ministry of Health, Czech Republic, for the development of the research organization No. 00023001 (IKEM, Prague, Czech Republic). We would like to thank researchers, interviewers and participants in Novosibirsk, Krakow, Kaunas, Havířov/Karviná, Jihlava, Ústí nad Labem, Liberec, Hradec Králové, and Kromĕříz.; **Inter99**: The Inter99 study was supported by the Danish Medical Research Council, the Danish Centre for Evaluation and Health Technology Assessment, Copenhagen County, the Danish Heart Foundation, the Danish Pharmaceutical Association, the Health Insurance Foundation, the Augustinus Foundation, the Ib Henriksens foundation and the Beckett Foundation. The present study was further supported by the Danish Diabetes Association (grant No. 32, December 2005) and the Health Insurance Foundation (grant No. 2010 B 131); **Izhevsk**: The Izhevsk Family Studies was funded by a UK Wellcome Trust programme grant (078557); **MESA**: The Multi-Ethnic Study of Atherosclerosis Study (MESA) is a multicenter prospective cohort study initiated to study the development of subclinical cardiovascular disease. A total of 6814 women and men between the age of 45 and 84 year were recruited for the first examination between 2000 and 2002. Participants were recruited in six US cities (Baltimore, MD; Chicago, IL; Forsyth County, NC; Los Angeles County, CA; Northern Manhattan, NY; and St. Paul, MN). This study was approved by the institutional review boards of each study site, and written informed consent was obtained from all participants. This cohort was genotyped as part of the National Heart Lung and Blood Institute’s (NHLBI) Candidate Gene Association Resource (CARe) (Musunuru, K., Lettre, G., Young, T., Farlow, D.N., Pirruccello, J.P., Ejebe, K.G., Keating, B.J., Yang, Q., Chen, M.H., Lapchyk, N. et al. Candidate gene association resource (CARe): design, methods, and proof of concept. Circ. Cardiovasc. Genet, 3, 267-275.); **NPHS II**: NPHS-II was supported by the British Medical Research Council, the US National Institutes of Health (grant NHLBI 33014), and Du Pont Pharma, Wilmington, Delaware; **Whitehall II**: The Whitehall II study and Mika Kivimaki were supported by the Medical Research Council; the British Heart Foundation; the Economic and Social Research Council; the National Heart Lung and Blood Institute (NHLBI: HL36310); and the National Institute on Aging (AG13196), US, NIH; **WHI**: The WHI program is funded by the National Heart, Lung, and Blood Institute, National Institutes of Health, U.S. Department of Health and Human Services through contracts HHSN268201100046C, HHSN268201100001C, HHSN268201100002C, HHSN268201100003C, HHSN268201100004C, and HHSN271201100004C. A listing of WHI investigators can be found at https://cleo.whi.org/researchers/Documents%20%20Write%20a%20Paper/WHI%20Investigator%20Short%20List.pdf.

## Alcohol-*ADH1B* Consortium

Michael V Holmes1, MRCP, MSc, Caroline E Dale2, PhD, Luisa Zuccolo3^,^4, PhD, Richard J Silverwood2, PhD, Yiran Guo5^,^6, PhD, Zheng Ye7, PhD, David Prieto-Merino2, PhD, Abbas Dehghan8, MD, PhD, Stella Trompet9, PhD, Andrew Wong^10^, Alana Cavadino^11^, MSc, Kieran McCaul^12^, PhD, Dagmar Drogan^13^, MPH, Sandosh Padmanabhan^14^, MBBS, PhD, Shanshan Li^15^, MD, MSc, Ajay Yesupriya^16^, MPH, Maarten Leusink^17^, MSc, Johan Sundstrom^18^, MD, PhD, Hynek Pikhart1, PhD, Daniel I Swerdlow1, PhD, Andrie G Panayiotou^19^, PhD, Svetlana A. Borinskaya^20^, PhD, Chris Finnan1, PhD, Sonia Shah^21^, MSc, Karoline B Kuchenbaecker^22^, Dipl.Pysch., MSc, Tina Shah1, PhD, Jorgen Engemann1, Lasse Folkerson^23^, PhD, Per Eriksson^23^, PhD, Fulvio Ricceri^24^, PhD, Olle Melander^25^, MD, PhD, Carlotta Sacerdote^26^, MD, PhD, Dale M Gamble^27^, MHSc, CCRP, Sruti Rayaprolu^28^, BSc, Owen A Ross^28^, PhD, Stela McLachlan^29^, PhD, Olga Vikhireva1, MD, PhD, Ivonne Sluijs^30^, PhD, Robert A Scott7, PhD, Vera Adamkova^31^, MD, Leon Flicker^12^, FRACP, PhD, Frank M van Bockxmeer^32^, FAHA, Pedro Marques-Vidal^33^, MD, PhD, Tom Meade2, FRS, Sir Michael G Marmot^34^, FRCP, PhD, Jose M Ferro^35,36^, MD, Sofia Paulos-Pinheiro^37,38^, Steve Humphries^39^, FRCPath, FRCP, PhD, Philippa Talmud^39^, FRCPath, DSc, Irene Mateo Leach^40^, PhD, Niek Verweij^40^, MSc, Allan Linneberg^41^, MD, PhD, Tea Skaaby^41^, MD, Pieter A Doevendans^42^, MD, PhD, Maarten J Cramer^42^, MD, PhD, Pim van der Harst^40, 43, 44^, MD, PhD, Olaf H Klungel^17^, PharmD, PhD, Nicole F Dowling^16^, PhD,Anna F Dominiczak^14^, OBE, FRCP, FRSE, FAHA, Meena Kumari1, PhD, Andrew Nicolaides^45,46,47^, FRCS, PhD, Shah Ebrahim2^, 48^, FRCP, DM, Tom R Gaunt4, PhD, Jackie F Price^29^, MD, Lars Lannfelt^49^, MD, PhD, Anne Peasey1, PhD, Anke H Maitland-van der Zee^17^, PhD, Paul E Norman^50^, MD, Graeme J Hankey^51, 52^, MD, FRACP, Manuela M Bergmann^13^, PhD, Albert Hofman8, MD, PhD, Oscar H Franco8, MD, PhD, Jackie Cooper^53^, MSc, Jutta Palmen^39^, PhD, Wilko Spiering^54^, MD, PhD, Pim de Jong^55^, MD, PhD, Diana Kuh^10^, PhD, Rebecca Hardy^10^, PhD, Andre G UItterlinden8, PhD, Arfan M Ikram8, MD, PhD, Ian Ford^56^, PhD, Elina Hypponen^11, 57^, PhD, Osvaldo Almeida^12, 58, 59^, MD, Nicholas J Wareham7, MB, PhD, Kay-Tee Khaw^60^, FRCP, PhD, Anders Hamsten^23, 61^, FRCP, PhD, on behalf of IMPROVE study group^62^, Lise Lotte N Husemoen^41^, PhD, Anne Tjønneland^63^, MD, PhD, Janne S Tolstrup^64^, PhD, Eric Rimm^15^, Sc.D., Jaroslav Hubacek^31^, DSc, PhD, Joline WJ Beulens^30^, PhD, WM Monique Verschuren^65^, PhD, N Charlotte Onland-Moret^30, 66^, PhD, Marten H Hofker^67^, PhD, S. Goya Wannamethee^68^, PhD, Peter H Whincup^69^, PhD, FRCP, Richard Morris^68^, PhD, Astrid M Vicente^37, 70, 68^, PhD, Hugh Watkins^72, 73^, FRCP, PhD, Martin Farrell^72, 73^, FRCPath, J Wouter Jukema9^, 42, 44^, MD, PhD, James Meschia^28^, MD, L Adrienne Cupples^74, 75^, PhD, Stephen J Sharp7, Msc, The InterAct Consortium^76^, Myriam Fornage^77^, PhD, Matthew B Lanktree^78^, BSc, David S. Siscovick^79^, MD, MPH, Eric Jorgenson^80^, PhD, Bonnie Spring^81^, PhD, Josef Coresh^82^, MD, PhD, MHS, Yun R Li5, BSc, Sarah G Buxbaum^83^, PhD, Pamela J Schreiner^84^, PhD, R Curtis Ellison^85^, MD, Michael Y Tsai^86^, MD, PhD, Sanjay R Patel^93^, MD, Susan Redline^15^, MD, MPH, Andrew D Johnson^75^, PhD, Ron C Hoogeveen^87^, PhD, Hakon Hakonarson4, MD, PhD, Jerome I. Rotter^88^, MD, Eric Boerwinkle^89^, PhD, Paul IW de Bakker^30, 90^, PhD, Mika Kivimaki1, PhD, Folkert Asselbergs^30, 42, 90^, MD, PhD, Naveed Sattar^91^, FRCP, Debbie Lawlor3^,4^, PhD, John Whittaker2^, 92^, PhD, George Davey Smith3^,4^, MD, DSc, Ken Mukamal^93^, MD, MPH, Bruce Psaty^79, 94^, MD, PhD, MPH, James G Wilson^95^, MD, PhD, Leslie A Lange^96^, PhD, Ajna Hamidovic^97^, PharmD, Aroon D Hingorani1, FRCP, PhD, Børge G Nordestgaard^98, 99, 100^, MD, DMSc, Martin Bobak1, MD, PhD, David Leon2, PhD, Claudia Langenberg7, MD, PhD, Tom Palmer^101^, PhD, Alex P Reiner^102^, MD, MSc, Brendan J Keating5, PhD, Frank Dudbridge2, PhD, Juan P Casas1^, 2^, MD, PhD

1. Genetic Epidemiology Group, Institute of Cardiovascular Science, Department of Epidemiology and Public Health, University College London, UK.
2. Faculty of Epidemiology and Population Health, London School of Hygiene & Tropical Medicine, London, UK.
3. MRC Integrative Epidemiology Unit, University of Bristol, Bristol, UK.
4. School of Social and Community Medicine, University of Bristol, Bristol, UK.
5. Center for Applied Genomics, Abramson Research Center, The Children's Hospital of Philadelphia, PA, USA.
6. BGI-Shenzhen, Beishan Industrial Zone, Yantian District, Shenzhen, China.
7. MRC Epidemiology Unit, Institute of Metabolic Science, Addenbrooke's Hospital, Cambridge, UK.
8. Department of Epidemiology, Erasmus Medical Center, Rotterdam, The Netherlands.
9. Department of Cardiology, Leiden University Medical Center, Leiden, The Netherlands.
10. MRC National Survey of Health and Development, MRC Unit for Lifelong Health and Ageing at UCL, London, UK.
11. Centre for Paediatric Epidemiology and Biostatistics, MRC Centre of Epidemiology for Child Health, UCL Institute of Child Health, London, UK.
12. Western Australian Centre for Health & Ageing, Centre for Medical Research, University of Western Australia, Perth, Western Australia, Australia.
13. German Institute of Human Nutrition Potsdam-Rehbrücke, Nuthetal, Germany.
14. Institute of Cardiovascular and Medical Sciences, College of Medical, Veterinary and Life Sciences, University of Glasgow, Glasgow, United Kingdom.
15. Department of Epidemiology, Harvard Medical School, Brigham and Women's Hospital, Boston, MA, USA.
16. Office of Public Health Genomics, Office of Epidemiology, Surveillance, and Laboratory Services, Centers for Disease Control and Prevention, Atlanta, GA, USA.
17. Division of Pharmacoepidemiology and Clinical Pharmacology, Utrecht Institute for Pharmaceutical Sciences, Utrecht University, Utrecht, The Netherlands.
18. Department of Medical Sciences, Uppsala University, Uppsala University Hospital, Uppsala, Sweden.
19. Cyprus International institute for Environmental and Public Health in association with the Harvard School of Public Health, Cyprus University of Technology, Limassol, Cyprus.
20. Vavilov Institute of General Genetics, Russian Academy of Sciences, Moscow, Russia.
21. UCL Genetics Institute, Department of Genetics Environment and Evolution, London, UK.
22. Centre for Cancer Genetic Epidemiology, Department of Public Health and Primary Care, University of Cambridge, Cambridge, UK.
23. Atherosclerosis Research Unit, Center for Molecular Medicine, Department of Medicine, Karolinska Institutet, Stockholm, Sweden.
24. HuGeF Foundation, Torino, Italy.
25. Department of Clinical Sciences, Lund University, Malmö, Sweden.
26. Unit of Cancer Epidemiology, San Giovanni Battista Hospital and Center for Cancer Prevention (CPO-Piemonte), Torino, Italy.
27. Mayo Clinic, Jacksonville, FL, USA.
28. Department of Neuroscience, Mayo Clinic Florida, Jacksonville, FL, USA.
29. Centre for Population Health Sciences, University of Edinburgh, Edinburgh, UK.
30. Julius Center for Health Sciences and Primary Care, University Medical Center, Utrecht, The Netherlands.
31. **Center for Experimental Medicine, Institute for Clinical and Experimental Medicine, Prague, Czech Republic**.
32. School of Pathology and Laboratory Medicine, the University of Western Australia.
33. Institute of Social and Preventive Medicine (IUMSP), CHUV and Faculty of Biology and Medicine, Lausanne, Switzerland.
34. UCL Institute of Health Equity, Department of Epidemiology & Public Health, London, UK.
35. Instituto Medicina Molecular, Faculdade de Medicina Universidade de Lisboa, Lisbon, Portugal.
36. Servico Neurologia, Hospital de Santa Maria, Lisbon, Portugal.
37. Instituto Nacional de Saude Doutor Ricardo Jorge, Lisbon, Portugal.
38. Faculdade Ciencias Universidade Lisboa, Campo Grande, Lisbon, Portugal.
39. Centre for Cardiovascular Genetics, Institute of Cardiovascular Science, University College London, London, UK.
40. Department of Cardiology, University Medical Center Groningen, Groningen, The Netherlands.
41. Research Centre for Prevention and Health, Capital Region of Denmark, Glostrup University Hospital, Glostrup, Denmark.
42. Department of Cardiology, Division Heart and Lungs, University Medical Center Utrecht, Utrecht, The Netherlands
43. Department of Genetics, University Medical Center Groningen, Groningen, The Netherlands.
44. Durrer Center for Cardiogenetic Research, Amsterdam, The Netherlands.
45. Vascular Screening and Diagnostic Centre, 2 Kyriakou Matsi str, Ayios Dometios, Nicosia, Cyprus.
46. Deparment of Vascular Surgery, Imperial College, London, UK.
47. Cyprus Cardiovascular Disease Educational and Research Trust, Nicosia, Cyprus.
48. South Asia Network for Chronic Disease, Public Health Foundation of India, New Delhi, India.
49. Department of Public Health & Caring Sciences, Uppsala University, Uppsala University Hospital, Uppsala, Sweden.
50. School of Surgery, University of Western Australia, Perth, Australia.
51. Stroke Unit, Department of Neurology, Royal Perth Hospital, 197 Wellington Street, Perth, Australia.
52. School of Medicine and Pharmacology, The University of Western Australia, Nedlands, Perth, Australia.
53. Centre for Cardiovascular Genetics, Institute of Cardiovascular Science, Rayne Building, University College London, London, UK.
54. Department of Vascular Medicine, University Medical Center Utrecht, Utrecht, The Netherlands.
55. Department of Radiology, University Medical Center Utrecht, Utrecht, The Netherlands.
56. Robertson Centre for Biostatistics, University of Glasgow, Glasgow, UK.
57. School of Population Health, University of South Australia, Adelaide, Australia.
58. School of Psychiatry & Clinical Neurosciences, University of Western Australia, Perth, Western Australia, Australia
59. Department of Psychiatry, Royal Perth Hospital, Perth, Western Australia, Australia.
60. Department of Primary Care and Public Health and Primary Care, University of Cambridge, Cambridge, UK.
61. Center for Molecular Medicine, Karolinska University Hospital Solna, Stockholm, Sweden.
62. The IMPROVE study group list of authors is as follows: D. Baldassarre (Dipartimento di Scienze Farmacologiche e Biomolecolari, Università di Milano, Milan, Italy and Centro Cardiologico Monzino, IRCCS, Milan Italy.); F. Veglia (Centro Cardiologico Monzino, IRCCS, Milan Italy); A. Hamsten (Atherosclerosis Research Unit, Department of Medicine Solna, Karolinska Institutet, Stockholm, Sweden.); S.E. Humphries ( British Heart Foundation Laboratories, University College of London, Department of Medicine, Rayne Building, London, United Kingdom.); R. Rauramaa (Foundation for Research in Health Exercise and Nutrition, Kuopio Research Institute of Exercise Medicine, Kuopio, Finland.); Ulf de Faire (Division of Cardiovascular Epidemiology, Institute of Environmental Medicine, Karolinska Institutet, and Department of Cardiology, Karolinska University Hospital, Solna, Karolinska Institutet, Stockholm, Sweden.); A.J. Smit ( Department of Medicine, University Medical Center Groningen, Groningen, the Netherlands.); P. Giral (Assistance Publique - Hopitaux de Paris; Service Endocrinologie-Metabolisme, Groupe Hôpitalier Pitie-Salpetriere, Unités de Prévention Cardiovasculaire, Paris, France.); S. Kurl (Institute of Public Health and Clinical Nutrition, University of Eastern Finland, Kuopio Campus.); E. Mannarino (Internal Medicine, Angiology and Arteriosclerosis Diseases, Department of Clinical and Experimental Medicine, University of Perugia, Perugia, Italy.); E. Grossi (Bracco Milan Italy.); R. Paoletti (Dipartimento di Scienze Farmacologiche e Biomolecolari, Università di Milano, Milan, Italy.); E. Tremoli (Dipartimento di Scienze Farmacologiche e Biomolecolari, Università di Milano, Milan, Italy and Centro Cardiologico Monzino, IRCCS, Milan Italy.)
63. Danish Cancer Society, Strandboulevarden, Copenhagen, Denmark.
64. National Institute of Public Health, University of Southern Denmark, Copenhagen, Denmark.
65. National Institute for Public Health and the Environment (RIVM), Bilthoven, The Netherlands.
66. Complex Genetics Section, Department of Medical Genetics (DBG), University Medical Center Utrecht, Utrecht, The Netherlands.
67. Dept. Pathology and Medical Biology, Medical Biology division, Molecular Genetics, University Medical Center Groningen and Groningen University, Groningen, The Netherlands.
68. Department of Primary Care & Population Health, UCL, Royal Free Campus, Rowland Hill St, London, UK.
69. Division of Population Health Sciences and Education, St George’s, University of London, London, UK.
70. Instituto Gulbenkian Ciencia, Oeiras, Portugal.
71. Biofig - Center for Biodiversity, Functional and Integrative Genomics, Campus da FCUL, Lisboa, Portugal.
72. Wellcome Trust Centre for Human Genetics, University of Oxford, Oxford, UK.
73. Department of Cardiovascular Medicine, University of Oxford, Oxford, UK.
74. Boston University, Boston, MA, USA.
75. National Heart, Lung, and Blood Institute's The Framingham Heart Study, Framingham, MA, USA.
76. The InterAct Consortium list of authors is as follows: C. Langenberg (MRC Epidemiology Unit, Cambridge, UK); S. Sharp (MRC Epidemiology Unit, Cambridge, UK); N.G. Forouhi (MRC Epidemiology Unit, Cambridge, UK); P.W. Franks (Lund University, Malmö, Sweden); M.B. Schulze (German Institute of Human Nutrition, Potsdam-Rehbruecke, Germany); N. Kerrison (MRC Epidemiology Unit, Cambridge, UK); U. Ekelund (MRC Epidemiology Unit, Cambridge, UK); I. Barroso (Wellcome Trust Sanger Institute, Cambridge, UK); S. Panico (Federico II University, Naples, Italy); M.J. Tormo (Department of Epidemiology, Murcia Regional Health Council, Murcia, Spain); J. Spranger (Charité University Berlin, Germany); S. Griffin (MRC Epidemiology Unit, Cambridge, UK); Y.T. van der Schouw (University Medical Center Utrecht, the Netherlands); P. Amiano (Public Health Division of Gipuzkoa, San Sebastian, Spain); E. Ardanaz (Navarre Public Health Institute, Pamplona, Spain); L. Arriola (Public Health Division of Gipuzkoa, San Sebastian, Spain); B. Balkau (INSERM, University Paris Sud, France); A. Barricarte (Epidemiology, Prevention and Promotion Health Service, Pamplona, Spain); J.W.J. Beulens (University Medical Center, Utrecht, the Netherlands); H. Boeing (German Institute of Human Nutrition, Potsdam-Rehbruecke, Germany); H.B. Bueno-de-Mesquita (National Institute for Public Health and the Environment, Bilthoven, the Netherlands); B. Buijsse (German Institute of Human Nutrition Potsdam-Rehbruecke, Germany); M.D. Chirlaque Lopez (Murcia Regional Health Authority, Spain); F. Clavel-Chapelon (INSERM, University Paris Sud, France); F.L. Crowe (University of Oxford, UK); B. de Lauzon-Guillan (INSERM, University Paris Sud, France); P. Deloukas (Wellcome Trust Sanger Institute, Cambridge, UK); M. Dorronsoro (Public Health Division of Gipuzkoa, San Sebastian, Spain); D. Drogan (German Institute of Human Nutrition, Potsdam-Rehbruecke, Germany); P. Froguel (Imperial College London, UK); C. Gonzalez (Catalan Institute of Oncology, Barcelona, Spain); S. Grioni (Fondazione IRCCS Istituto Nazionale Tumori, Milan, Italy); L. Groop (University Hospital Scania, Malmö, Sweden); C. Groves (University of Oxford, UK); P. Hainaut (International Agency for Research of Cancer, Lyon, France); J. Halkjaer (Danish Cancer Society, Copenhagen, Denmark); G. Hallmans (Umea University, Sweden); T. Hansen (Hagedorn Research Institute, Copenhagen, Denmark); J.M. Huerta Castaño (Murcia Regional Health Authority, Spain); R. Kaaks (German Cancer Research Centre, Heidelberg, Germany); T.J. Key (University of Oxford, UK); K.T. Khaw (University of Cambridge, UK); A. Koulman (MRC Human Nutrition Research, Cambridge, UK); A. Mattiello (Federico II University, Naples, Italy); C. Navarro (Murcia Regional Health Authority, Spain); P. Nilsson (Lund University, Malmö, Sweden); T. Norat (Imperial College London, UK); K. Overvad (School of Public Health, Aarhus, Denmark); L. Palla (MRC Epidemiology Unit, Cambridge, UK); D. Palli (Cancer Research and Prevention Institute (ISPO), Florence, Italy); O. Pedersen (Hagedorn Research Institute, Copenhagen, Denmark); P.H. Peeters (University Medical Center Utrecht, the Netherlands); J.R. Quirós (Asturias Health and Health Care Council, Oviedo, Spain); A. Ramachandran (India Diabetes Research Foundation, Chennai, India); L. Rodriguez-Suarez (Asturias Health and Health Care Council, Oviedo, Spain); O. Rolandsson (Umea University, Sweden); D. Romaguera (Imperial College London, UK); I. Romieu (International Agency for Research of Cancer, Lyon, France); C. Sacerdote (Center for Cancer Prevention, Torino, Italy); M.J. Sánchez (Andalusian School of Public Health, Granada, Spain); A. Sandbaek (School of Public Health, Aarhus, Denmark); N. Slimani (International Agency for Research of Cancer, Lyon, France); I. Sluijs (University Medical Center, Utrecht, the Netherlands); A.M.W. Spijkerman (National Institute for Public Health and the Environment, Bilthoven, the Netherlands); B. Teucher (German Cancer Research Centre, Heidelberg, Germany); A. Tjonneland (Danish Cancer Society, Copenhagen, Denmark); R. Tumino (Cancer Registry and Histopathology Unit, Ragusa, Italy); D.L. van der A (National Institute for Public Health and the Environment, Bilthoven, the Netherlands); W.M.M. Verschuren (National Institute for Public Health and the Environment, Bilthoven, the Netherlands); J. Tuomilehto (University of Helsinki, Finland); E. Feskens (University of Wageningen, the Netherlands); M. McCarthy (University of Oxford, UK); E. Riboli (Imperial College London, UK); N.J. Wareham (MRC Epidemiology Unit, Cambridge, UK).
77. Institute of Molecular Medicine, The University of Texas Health Science Center at Houston, Utah, USA.
78. Schulich School of Medicine and Dentistry, University of Western Ontario, London, ON, Canada.
79. Cardiovascular Health Research Unit, Departments of Medicine, Epidemiology, and Health Services, University of Washington, Seattle, WA,USA.
80. University of California San Francisco, CA, USA.
81. National Heart, Lung and Blood Institute Bethesda, MD, USA.
82. Department of Epidemiology, Johns Hopkins Bloomberg School of Public Health, Johns Hopkins University, Baltimore, MD.
83. Jackson Heart Study, Jackson State University, Jackson, MS, USA; School of Health Sciences, Department of Epidemiology and Biostatistics, Jackson State University, Jackson, MS, USA.
84. School of Public Health, University of Minnesota, Minneapolis, Minnesota, USA.
85. Preventive Medicine and Epidemiology, Evans Department of Medicine, Boston University School of Medicine, Boston, Massachusetts, USA.
86. Department of Laboratory Medicine and Pathology, University of Minnesota, USA.
87. Baylor College of Medicine, Department of Medicine, Division of Atherosclerosis & Vascular Medicine, Houston, Texas, USA.
88. Medical Genetics Institute, Department of Medicine, Cedars-Sinai, Los Angeles, USA.
89. Division of Epidemiology, School of Public Health, The University of Texas Health Science Center at Houston, Utah, USA.
90. Department of Medical Genetics, Biomedical Genetics, University Medical Center, Utrecht, The Netherlands.
91. British Heart Foundation Glasgow Cardiovascular Research Centre, University of Glasgow, Glasgow, UK.
92. Genetics, R&D, GlaxoSmithKline, Stevenage, UK.
93. Beth Israel Deaconess Medical Center, Boston, MA, USA.
94. Group Health Research Institute, Group Health Cooperative, Seattle, WA, USA.
95. Department of Physiology and Biophysics, University of Mississippi Medical Center, Jackson, MS, USA.
96. Department of Genetics, University of North Carolina School of Medicine at Chapel Hill, Chapel Hill, North Carolina, USA.
97. College of Pharmacy, The University of New Mexico, Albuquerque, NM, USA.
98. The Copenhagen General Population Study, Herlev Hospital, Copenhagen, Denmark.
99. Faculty of Health Sciences, Copenhagen University Hospital, University of Copenhagen, Copenhagen, Denmark.
100. Department of Clinical Biochemistry, Herlev Hospital, Copenhagen University Hospital, Denmark.
101. Division of Health Sciences, Warwick Medical School, University of Warwick, Coventry, UK.
102. Division of Public Health Sciences, Fred Hutchinson Cancer Research Center, Seattle, WA, USA.
