# Supplementary material for: Testing for non-linear causal effects using a binary genotype in a Mendelian randomization study: application to alcohol and cardiovascular traits
Source: Int J Epidemiol. 2014 Sep 5;43(6):1781–90. doi: 10.1093/ije/dyu187 (PMC4276061; doi:10.1093/ije/dyu187)
Supplement: Supplementary Data [file supp_dyu187_ije-2013-11-1139-File005.docx]

# Testing for non-linear causal effects using a binary genotype in a Mendelian randomisation study: application to alcohol and cardiovascular traits

Richard J Silverwood, Michael V Holmes, Caroline E Dale, Debbie A Lawlor, John C Whittaker, George Davey Smith, David A Leon, Tom Palmer, Brendan J Keating, Luisa Zuccolo, Juan P Casas, Frank Dudbridge, on behalf of the Alcohol-*ADH1B* Consortium

## Supplementary material

### Simulations – Methods

We conducted simulations to assess the proposed approach in terms of bias and coverage at a variety of sample sizes and under different *X-Y* associations. We performed additional simulations to explore the effects of $\beta_{XG}$ heterogeneity and *G*-*U* interaction at both the individual and subgroup level.

The simulation parameters were chosen to resemble those observed in the Alcohol-*ADH1B* Consortium, and a plausible degree of confounding was applied. In each simulation the data were generated according to

$$Y\sim N(\beta_{1}X+\beta_{2}X^{2}-2U+U^{2}, 5^{2})$$

with the following specifications remaining constant:

$$G\sim\text{Bin}(1, 0.3); U\sim N(0, 1)$$

Thus, we allow for a quadratic causal effect of *X* on *Y*, but also for quadratic effects of confounders. The following simulations were conducted:

1. *Sample size*. The effect of sample size was examined using the null model with $(\beta_{1}, \beta_{2})=(0, 0)$. Sample size was varied within $\{5000, 10000, 50000, 100000\}$. *X* was simulated using $X\sim N(-0.2G+0.5U, 1)$.
2. *X-Y associations.* A variety of *X*-*Y* associations were explored using a sample size of 10000. Linear associations were generated using $(\beta_{1}, \beta_{2})=\{(-5, 0), (-2, 0), (-1, 0), (1, 0), (2, 0), (5, 0)\}$; quadratic associations were generated using $(\beta_{1}, \beta_{2})=\{(2, -5), (1, -2), (2, -1), (-2, 1), (-1, 2), (-2, 5)\}$. *X* was again simulated using $X\sim N(-0.2G+0.5U, 1)$.
3. *Individual-level* $\beta_{XG}$ *heterogeneity*. The key assumption of our approach is that $\beta_{XG}$ is constant for all individuals. The degree of variability introduced into these simulations regarding individual-level $\beta_{XG}$ heterogeneity was informed by the observation that within the *ADH1B* Consortium the study-specific estimates of the association between *rs1229984* and log-alcohol had mean -0.235 and SD 0.121, along with the assumption that individual-level heterogeneity is likely to be somewhat greater than this. We defined *X* using $X\sim N((-0.2+\alpha)G+0.5U, 1)$where $\alpha\sim N(0, \gamma^{2})$ was simulated independently for each subject within a sample size of 10,000 and $\gamma$ varied within $\{0, 0.1, 0.2, 0.3, 0.5, 1\}$. Thus, for $\gamma=0.1$ $\beta_{XG}$ is expected to lie between -0.4 and 0 in 95% of subjects, and approximately 61% of subjects are expected to be compliers in the sense that they are moved by the instrument to the adjacent bin of size$\beta_{XG}=-0.2$. Simulations were conducted using the quadratic data generating model with $(\beta_{1}, \beta_{2})=(-1, 2)$.
4. *Individual-level* $G$*-*$U$ *interaction*. We defined *X* using $X\sim N(-0.2G+0.5U+\delta GU, 1)$where $\delta$varied within $\{0, 0.1, 0.2, 0.3, 0.5, 1\}$in a sample of size 10,000. This means that the effect of *G* on *X* is allowed to vary by levels of the confounder *U*. Simulations were conducted using the quadratic data generating model with $(\beta_{1}, \beta_{2})=(-1, 2)$.
5. $\beta_{XG}$ *heterogeneity between unknown subgroups.* The degree of variability introduced into these and subsequent simulations regarding $\beta_{XG}$ heterogeneity between known or unknown subgroups were informed by the observation that within the *ADH1B* Consortium the study-specific estimates of the association between *rs1229984* and log-alcohol had mean -0.235 and SD 0.121. We defined *X* using $X\sim N((-0.2+\alpha)G+0.5U, 1)$where $\alpha\sim N(0, \gamma^{2})$ took the same value within subgroups of 1000 subjects within an overall sample size of 10,000 and $\gamma$ varied within $\{0, 0.01, 0.02, 0.03, 0.05, 0.1\}$. Thus, for $\gamma=0.1$ $\beta_{XG}$ is expected to lie between --0.4 and 0.0 in 95% of subgroups. Simulations were conducted using the quadratic data generating model with $(\beta_{1}, \beta_{2})=(-1, 2)$. Data were analysed as previously described, with all subgroups pooled together.
6. $\beta_{XG}$ *heterogeneity between known subgroups. X* was again defined using $X\sim N((-0.2+\alpha)G+0.5U, 1)$where $\alpha\sim N(0, \gamma^{2})$ now took the same value within subgroups of 10,000 subjects within an overall sample size of 100,000 and $\gamma$ varied between $\{0, 0.01, 0.02, 0.03, 0.05, 0.1\}$. Simulations were again conducted using the quadratic data generating model with $(\beta_{1}, \beta_{2})=(-1, 2)$. Data were analysed with all subgroups pooled together and adjustment for subgroup using indicator variables..
7. *G*-*U* *interaction which varies between unknown subgroups.* In addition to individual-level *G*-*U* interaction, the degree of interaction may also vary between subgroups in a population. We investigated this issue in the situation where the subgroups between which the *G*-*U* interaction varied are unknown. We defined *X* using $X\sim N(-0.2G+0.5U+\delta GU, 1)$where $\delta\sim N(0, \gamma^{2})$ took the same value within subgroups of 1000 subjects within an overall sample size of 10,000 and $\gamma$ varied within $\{0, 0.01, 0.02, 0.03, 0.05, 0.1\}$. Simulations were conducted using the quadratic data generating model with $(\beta_{1}, \beta_{2})=(-1, 2)$. Analysis proceeded as previously described, with data from all subgroups pooled together.

When regressing *Y* on $X^{*}$ we only estimated a LATE if there were at least 5 subjects with $X_{i}^{*}=j$ and $G_{i}=1$ and 5 subjects with $X_{i}^{*}=j-1$ and $G_{i}=0$, as regressions using smaller numbers of subjects were found to lead to anomalous estimated LATEs with spuriously high precision. A thousand simulations of each specification were conducted, with percentile bootstrap confidence intervals derived using 1000 bootstrap samples within each simulation. Standard 2SLS and quadratic OLS estimates were also calculated for comparison.

The analysis was conducted using R version 2.13.

### Simulations - Results

1. *Sample size*. Results for varying sample size are presented in Figure S1. The linear IV estimate, mean LATE, LATE intercept and LATE slope were unbiased even with a sample size of 5000. The variability in the mean LATE and LATE slope was similar to that of the linear IV estimate. The quadratic OLS estimates were significantly biased at all sample sizes. The corresponding coverages are shown in Figure S2. The linear IV estimate, mean LATE and LATE intercept coverages varied between 94% and 96%, even at smaller sample sizes. The coverage of the LATE slope was somewhat conservative (around 97%) at smaller sample sizes.
2. *X-Y associations.* In the linear data generating models the linear IV estimate, mean LATE, LATE intercept and LATE slope were all unbiased (Figure S3). The mean LATE and LATE slope again had similar variability to the linear IV estimate. The quadratic OLS estimates were biased in all scenarios. The coverages of the linear IV estimate, mean LATE, and LATE intercept were generally between 94% and 96%, but the coverage of the LATE slope was approximately 96% across the range of linear coefficients (Figure S4). This suggests that our procedure gives a slightly conservative test of no non-linear effect. In the quadratic data generating models the LATE intercepts and LATE slopes were unbiased for all combinations of coefficients but the quadratic OLS estimates were again heavily biased (Figure S5). The coverages of the LATE intercept and LATE slope were generally between 94% and 96%, though the LATE slope coverage was as high as 97.5% for $(\beta_{1}, \beta_{2})=(-2, 5)$ (Figure S6).
3. *Individual-level* $\beta_{XG}$ *heterogeneity*. For small values of $\gamma$ the LATE intercept and LATE slope displayed little bias (Figure S7). However, for values of $\gamma$of 0.2 and greater noticeable bias began to appear, particularly in the LATE slope. By $\gamma=1$ the bias was so great that the 95% range of estimates of the LATE slope did not include the target value. Both the LATE intercept and LATE slope had acceptable levels of coverage for values of $\gamma$ up to 0.3 (Figure S8). For values of $\gamma$ larger than 0.5 the coverage of the LATE slope was drastically reduced. However, it should be noted that the larger values of $\gamma$ in these simulations do represent very extreme cases (e.g. for $\gamma=1$ $\beta_{XG}$ is expected to lie between -2.2 and 1.8 in 95% of subjects, so in almost 50% of subjects the gene would have an effect on alcohol consumption in the opposite direction to that assumed). Thus, given the reliance of our method on the assumption of $\beta_{XG}$ homogeneity, the observed bias and reduced coverage is not unexpected.
4. *Individual-level* $G$*-*$U$ *interaction*. Bias was observed for all values of $\delta$ greater than zero for both the LATE intercept and the LATE slope (Figure S9). The bias in the LATE intercept increased as $\delta$ increased, but the bias in the LATE slope decreased slightly at $\delta=1$. The coverage of the parameters correspondingly generally deviated further from 95% as $\delta$increased (Figure S10), reaching 74% for the LATE intercept and 59% for the LATE slope at $\delta=0.5$. Coverage for the LATE intercept reduced further to 14% at $\delta=1$, but improved slightly for the LATE slope.
5. $\beta_{XG}$ *heterogeneity between unknown subgroups.* At no values of $\gamma$ was there any appreciable bias in either the LATE intercept or the LATE slope (Figure S11). For all values of $\gamma$ the LATE intercept had coverage close to 95%, but there was again some evidence that the coverage of the LATE slope was slightly conservative (Figure S12).
6. $\beta_{XG}$ *heterogeneity between known subgroups.*  At no values of $\gamma$ was there any evidence of bias in either the LATE intercept or the LATE slope (Figure S13). Coverage was approximately 96% for the LATE intercept and 97-99% for the LATE slope (Figure S14).
7. *G-U*  *interaction which varies between unknown subgroups.* There was no bias in either parameter at $\gamma=0.1$, but bias in both parameters, most noticeably in the LATE slope, increased as $\gamma$ increased further (Figure S15). Coverage was approximately appropriate for the LATE intercept as far as $\gamma=0.5$, though it was <90% for the LATE slope from $\gamma=0.3$ onwards (Figure S16). By $\gamma=1$ the coverages of the LATE intercept and slope were 84% and 23% respectively.

These results suggest that the LATE estimates are essentially unbiased with generally good coverage properties under null, linear and quadratic models. Reasonable levels of individual-level heterogeneity in $\beta_{XG}$ were not found to lead to significant bias in the estimates. Low levels of between-subgroup heterogeneity in $\beta_{XG}$ were also not found to lead to significant bias, whether or not the heterogeneity was adjusted for in the analysis. High levels of interaction between $G$ and $U$ led to bias in the estimates, but such interactions may be unlikely in practice. For example, in simulation d) a $\delta$ of 0.2 biased the LATE intercept upwards by 30% and the LATE slope upwards by 16%. This level of interaction means that the effect of $G$ on $X$can be expressed as -0.2 + 0.2$U$. As $U\sim N(0, 1)$, 95% of the values of $U$will lie between -2 and 2 (approximately). Thus the effect of $G$ on $X$will lie between -0.6 and 0.2 approximately 95% of the time, depending on the value of $U$. Such a wide range of genetic effects, including a reversal of sign, may well be deemed implausible.

Overall, our simulations indicate that the LATE method is a useful extension to standard approaches in the non-linear setting.

### Multiple studies

When data from multiple studies are available we must decide whether to estimate LATEs within each study, combining them to draw an overall conclusion, or to estimate LATEs on the combined data. In general, weak instrument bias is reduced by performing the MR analysis on the combined data. But because our approach relies on the genetic effect size $\beta_{XG}$ being constant for all individuals, we need to consider whether this assumption is tenable across studies as well as within each study.

If $\beta_{XG}$ varies across studies then clearly we should perform the procedure separately in each study. This however raises two problems. Firstly, the restriction to subjects having particular genotype-exposure combinations in each bin of $X^{*}$ leads to small sample sizes for estimating some LATEs, leading to large standard errors on some $\hat{\tau}_{j}$ and considerable uncertainty on the final inference of non-linearity. This problem occurs particularly when one genotype is rare, or when $\beta_{XG}$ is small, leading to narrow bin definitions, both of which apply to the Alcohol-*ADH1B* Consortium. Secondly, different $\beta_{XG}$ across studies leads to different bin sizes across studies and different local causal effects being estimated. It is not clear how such effects should be combined into an overall inference on non-linearity.

For these reasons we assumed that $\beta_{XG}$ is constant within and between studies and performed simulations to assess robustness to that assumption. As in the standard IV analysis, covariates for study, age and sex were included in both the estimation of $\beta_{XG}$ and the LATEs in order to reduce the potential for confounding of $\beta_{XG}$ and the LATEs.

Table S1. Design and genotyping characteristics of the studies included in the analysis.

| Study | Study design | Sampling Frame | Number with DNA in this analysis | Number contributing to one or more of our analyses^A^ | Year of blood sampling used for DNA extraction | Genotyping method | Country | HWE P value (exact significance probability) | Call rate (%) |
| --- | --- | --- | --- | --- | --- | --- | --- | --- | --- |
| ARIC | Cohort | Community | 9557 | 9532 | 1987-89 | IBC 50k CardioChip | USA | 0.705 | 97.8 |
| BRHS | Cohort | General practices | 3843 | 3789 | 1998-2000 | KASPar | UK | 0.42 | 100 |
| BWHHS | Cohort | General practices | 3412 | 3407 | 1999-2001 | Illumina HumanCVD array | UK | 0.912 | 99.7 |
| CaPS | Cohort | Electoral register & General practices | 1102 | 1061 | 1993-1994 | KASPar | UK | 0.460 | 98.4 |
| CARDIA | Cohort | Community | 1433 | 1433 | 1995-1996 | IBC 50k CardioChip | USA | 4.97E-04 | 97.3 |
| CCHS | Cohort | Population | 9081 | 8985 | 1991-94 | Nanogen | Denmark | 0.522 | 99.6 |
| CHS | Cohort | Community | 3936 | 3919 | 1992–1993 | IBC 50k CardioChip | USA | 0.001 | 97.9 |
| CYPRUS | Cohort | Community | 730 | 729 | 2003-2008 | TaqMan | Cyprus | 0.081 | 99.9 |
| Czech post-MONICA | Cohort | Administrative districts | 2558 | 2555 | 2000-2001 | PCR-RFLP | Czech Republic | 0.801 | 97.9 |
| DCH | Nested case cohort | General population (born in Denmark) | 2736 | 2735 | 1993-97 | TaqMan | Denmark | 0.203 | 91.8 |
| EAS | Cohort | General practices | 873 | 873 | 2004 | TaqMan | UK | 0.693 | 95.6 |
| ELSA | Cohort | Respondents of HSE | 5450 | 5449 | 2004 | KASPar | UK | 0.263 | 98.8 |
| EPIC Turin | Cohort | Population (Torino area) | 4526 | 4314 | 2008 | TaqMan | Italy | 0.362 | 99 |
| FHS | Cohort | Community | 1082 | 312 | 1948-present | IBC 50k CardioChip | USA | 0.002 | 99 |
| HAPIEE Czech | Cohort | City districts | 6678 | 6553 | 2003-2005 | KASPar | Czech Republic | 0.745 | 98.6 |
| Inter99 | RCT | Population | 6332 | 6025 | 1999-2001 | KASPar | Denmark | 6.16E-27 | 97.6 |
| Izhevsk | Case control | Population-based controls from CC | 653 | 642 | 2008-2009 | PCR + electrophoresis | Russia | 0.192 | >99 |
| MESA | Cohort | Population | 2293 | 2054 | 2000-2002 | IBC 50k CardioChip | USA | 0.012 | 97 |
| NPHS II | Cohort | General practices | 2659 | 2659 | 2000 | TaqMan | UK | 0.874 | 96.1 |
| ULSAM | Cohort | General population (Uppsala County) | 453 | 421 | 2004 | Illumina Golden Gate | Sweden | 0.775 | 98.91 |
| Whitehall II | Cohort | Workplace (civil servants) | 5029 | 4990 | 2002-2004 | IBC 50k CardioChip | UK | 0.106 | 99.3 |
| WHI | Nested case control | Community | 7882 | 7620 | 1993-1998 | IBC 50k CardioChip | USA | 3.15E-25 | 99.2 |

^A^Number of study members non-missing for weekly volume of alcohol, *rs1229984* polymorphism in *ADH1B*, age, sex, and one or more of the outcomes (systolic blood pressure, non-high density lipoprotein cholesterol, high density lipoprotein cholesterol, body mass index, waist circumference, C-reactive protein, interleukin 6 and triglycerides).

Figure S1. Observational quadratic model linear parameter estimates (brown) and quadratic parameter estimate (purple), two-stage least squares estimates (red), mean local average treatment effects (LATEs) (blue), LATE intercepts (green) and LATE slopes (orange) for different sample sizes in the null data generating model. Points represent means and bars represent 95% of the data. Horizontal solid lines represent target values.

Figure S2. 95% confidence interval coverage for the two-stage least squares estimates (red), mean LATEs (blue), LATE intercepts (green) and LATE slopes (orange) for different sample sizes in the null data generating model. Horizontal solid line represents the target value (95%).

Figure S3. Observational quadratic model linear parameter estimates (brown) and quadratic parameter estimate (purple), two-stage least squares estimates (red), mean LATEs (blue), LATE intercepts (green) and LATE slopes (orange) for different linear coefficients in the linear data generating model. Points represent means and bars represent 95% of the data. Horizontal solid lines represent target values.

Figure S4. 95% confidence interval coverage for the two-stage least squares estimates (red), mean LATEs (blue), LATE intercepts (green) and LATE slopes (orange) for different linear coefficients in the linear data generating model. Horizontal solid line represents the target value (95%).

**Figure S5. Observational quadratic model** **linear parameter estimates (brown) and quadratic parameter estimate (purple), two-stage least squares estimates (red), mean LATEs (blue), LATE intercepts (green) and LATE slopes (orange) for different combinations of linear and quadratic coefficients in the quadratic data generating model. Points represent means and bars represent 95% of the data. Horizontal solid lines represent target values.**

**Figure S6. 95% confidence interval coverage for the LATE intercepts (green) and LATE slopes (orange) for different combinations of linear and quadratic coefficients in the quadratic data generating model. Horizontal solid line represents the target value (95%).**

**Figure S7. LATE intercepts (green) and LATE slopes (orange) for different degrees of individual-level heterogeneity of $\boldsymbol{\beta}_{\boldsymbol{xg}}$ in the quadratic data generating model with linear coefficient = -1 and quadratic coefficient = 2. Points represent means and bars represent 95% of the data. Horizontal solid lines represent target values.**

**Figure S8. 95% confidence interval coverage for LATE intercepts (green) and LATE slopes (orange) for different degrees of individual-level heterogeneity of $\boldsymbol{\beta}_{\boldsymbol{xg}}$ in the quadratic data generating model with linear coefficient = -1 and quadratic coefficient = 2. Horizontal solid line represents the target value (95%).**

**Figure S9. LATE intercepts (green) and LATE slopes (orange) for different degrees of individual-level G-U interaction in the quadratic data generating model with linear coefficient = -1 and quadratic coefficient = 2. Points represent means and bars represent 95% of the data. Horizontal solid lines represent target values.**

**Figure S10. 95% confidence interval coverage for LATE intercepts (green) and LATE slopes (orange) for different degrees of individual-level G-U interaction in the quadratic data generating model with linear coefficient = -1 and quadratic coefficient = 2. Horizontal solid line represents the target value (95%).**

**Figure S11. LATE intercepts (green) and LATE slopes (orange) for different degrees of heterogeneity of $\boldsymbol{\beta}_{\boldsymbol{xg}}$ in unknown subgroups in the quadratic data generating model with linear coefficient = -1 and quadratic coefficient = 2. Points represent means and bars represent 95% of the data. Horizontal solid lines represent target values.**

**Figure S12. 95% confidence interval coverage for LATE intercepts (green) and LATE slopes (orange) for different degrees of heterogeneity of $\boldsymbol{\beta}_{\boldsymbol{xg}}$ in unknown subgroups in the quadratic data generating model with linear coefficient = -1 and quadratic coefficient = 2. Horizontal solid line represents the target value (95%).**

**Figure S13. LATE intercepts (green) and LATE slopes (orange) for different degrees of heterogeneity of $\boldsymbol{\beta}_{\boldsymbol{xg}}$ in known subgroups in the quadratic data generating model with linear coefficient = -1 and quadratic coefficient = 2. Points represent means and bars represent 95% of the data. Horizontal solid lines represent target values.**

**Figure S14. 95% confidence interval coverage for LATE intercepts (green) and LATE slopes (orange) for different degrees of heterogeneity of $\boldsymbol{\beta}_{\boldsymbol{xg}}$ in known subgroups in the quadratic data generating model with linear coefficient = -1 and quadratic coefficient = 2. Horizontal solid line represents the target value (95%).**

**Figure S15. LATE intercepts (green) and LATE slopes (orange) for different degrees of G-U interaction which varies between unknown subgroups in the quadratic data generating model with linear coefficient = -1 and quadratic coefficient = 2. Points represent means and bars represent 95% of the data. Horizontal solid lines represent target values.**

**Figure S16. 95% confidence interval coverage for LATE intercepts (green) and LATE slopes (orange) for different degrees of G-U interaction which varies between unknown subgroups in the quadratic data generating model with linear coefficient = -1 and quadratic coefficient = 2. Horizontal solid line represents the target value (95%).**

**
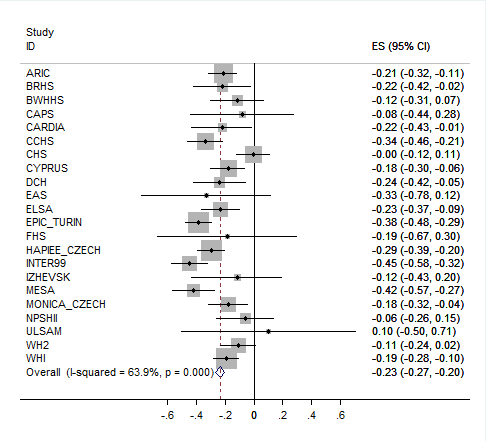
**

**Figure S17. Study-specific estimates of the association between *rs1229984* and log-alcohol.**

## References

1. R Development Core Team. R: A language and environment for statistical computing. Vienna, Austria, R Foundation for Statistical Computing. 2011.
2. Burgess S, Thompson SG, CRP CHD Genetics Collaboration. Avoiding bias from weak instruments in Mendelian randomization studies. *Int J Epidemiol* 2011; **40**: 755-64.
